# Designing an integrated data model for prospective genotype-phenotype in inborn errors of immunity research

**DOI:** 10.3389/fimmu.2026.1838940

**Published:** 2026-07-22

**Authors:** Maram Ahmed, Ahmed Aziz Bousfiha, Farida Almarzooqi

**Affiliations:** 1 Department of Internal Medicine, College of Medicine and Health Sciences, United Arab Emirates University, Al Ain, United Arab Emirates; 2 Pediatric Infectious and Immunological Diseases, Ibn Rochd University Hospital, Casablanca, Morocco; 3 Laboratory of Clinical Immunology, Infection and Autoimmunity (LICIA), Faculty of Medicine and Pharmacy, Hassan II University, Casablanca, Morocco; 4 Consultant Allergy and Clinical Immunology, Department of Internal Medicine, Tawam Hospital and Sheikh Tahnoun Bin Mohammed Medical City (STMC), Al Ain, United Arab Emirates

**Keywords:** data management, genotype, inborn errors of immunity, phenotype, REDCap, United Arab Emirates

## Abstract

**Background:**

Inborn errors of immunity are rare, genetically heterogeneous disorders requiring coordinated clinical, laboratory, and genetic evaluation over time. Data are often fragmented across records, laboratory systems, and genomic reports, limiting longitudinal analysis and coordinated care, particularly in the Middle East and North Africa, where structured rare disease data infrastructures remain limited.

**Objective:**

To develop a Research Electronic Data Capture–based data management framework for inborn errors of immunity and demonstrate its use in a prospective multi-site setting.

**Methods:**

A Research Electronic Data Capture–based framework was developed at the College of Medicine and Health Sciences, United Arab Emirates University. Modular instruments captured consent, demographics, biospecimen processing, laboratory workflows, and genetic findings within a longitudinal structure. Data dictionaries, validation rules, and conditional logic ensured data quality. The framework was deployed across participating sites for prospective data collection.

**Results:**

The framework enabled integrated longitudinal documentation of enrollment, biospecimens, and genetic testing. It was implemented across two clinical sites and used to enroll patients with suspected or confirmed inborn errors of immunity. The platform supported standardized cross-site data capture and monitoring of genetic findings, including automated flagging of variants of uncertain significance.

**Conclusion:**

This study demonstrates the development and early multi-site implementation of a Research Electronic Data Capture–based framework for inborn errors of immunity. By enabling standardized integration of clinical, laboratory, and genetic data, the platform supports data quality, cross-site collaboration, and tracking of evolving diagnoses. It provides a scalable foundation for rare disease research and may support improved clinical decision-making.

## 1 Introduction

Inborn errors of immunity (IEI) comprise a heterogeneous group of rare disorders characterized by evolving phenotypes, complex diagnostic pathways, and rapidly expanding genomic insight (1). The clinical course of IEI often unfolds over many years, requiring coordinated clinical, laboratory, and genetic evaluation to establish diagnosis, guide management, and monitor outcomes (2). Consequently, substantial volumes of clinical and genomic information accumulate across multiple patient encounters. To manage this complexity, structured data management systems have become increasingly important in rare disease research. Electronic data capture platforms such as Research Electronic Data Capture (REDCap), OpenClinica, and Castor EDC are widely used to support standardized data entry and longitudinal documentation (3–6). However, in many healthcare settings, information generated during the diagnostic process remains dispersed across clinical, laboratory, and research systems, limiting continuity of care, standardization, and long-term follow-up.

These challenges are particularly relevant in the Middle East and North Africa (MENA) region, where rare and genetically complex diseases are more prevalent due to high rates of consanguinity (7). Patients with suspected IEI often require specialized expertise concentrated in a limited number of centers, while clinical care and diagnostic evaluation frequently occur across multiple healthcare institutions. This fragmentation complicates systematic case aggregation, coordinated data collection, and follow-up. Structured data management frameworks are therefore essential to support harmonized documentation across sites and facilitate consolidation of clinical and genetic information for collaborative analysis. Databases such as the United States Immunodeficiency Network (USIDNET), and the European Society for Immunodeficiencies (ESID) registry and the recently developed Genetic Immunology Advisor (GenIA) platform demonstrate the value of coordinated datasets in advancing IEI research (8–10). However, establishing infrastructure for sustained data capture remains challenging, as systems developed in one healthcare setting are not always easily transferable to others due to differences in electronic health record systems, regulatory frameworks, and care delivery models (8, 9). Regional initiatives such as the MENAT-IEI Research Consortium Registry (MENAT-IEI-RCR) are beginning to emerge, reflecting growing recognition of the importance of collaborative IEI data infrastructure across the region (11).

Complementing these efforts, the need for interoperable data systems is increasingly evident in the United Arab Emirates, where expanding genomic testing capacity and growing recognition of IEI highlight the importance of platforms capable of integrating clinical and genetic information across institutions. Despite these advances, practical frameworks for standardized rare disease documentation remain limited. The success of national initiatives such as the UAE National Cancer Registry demonstrates the feasibility and value of coordinated disease data systems, providing a foundation for developing similar registries for rare and genetically complex disorders (12–14). To address this gap, we developed a REDCap^®^-based multi-site data management tool at the College of Medicine and Health Sciences, United Arab Emirates University, to enable standardized capture of clinical and genetic data for suspected or confirmed IEI and applied it across participating clinical sites for systematic patient data collection and integration. This perspective outlines the tool’s development and its application in multi-site clinical research.

## 2 Methods

### 2.1 Tool development: REDCap data management framework

#### 2.1.1 Study design and platform selection

A prospective, scalable data management tool for IEI was developed using the REDCap^®^ platform, a secure web-based electronic data capture system widely used in clinical and translational research. The platform is centrally hosted at United Arab Emirates University (redcap.uaeu.ac.ae), which serves as the coordinating hub for participating centers. The framework was established on September 4, 2025, with the initial international research collaboration with King Hassan II University in Casablanca, Morocco.

REDCap was selected for its flexible instrument design, secure data management infrastructure, role-based access control, and support for longitudinal data collection across multiple sites. This enables standardized capture of demographic, clinical, laboratory, and genetic data and facilitates harmonized multi-center research in rare disease cohorts such as IEI. To ensure data security and governance, the system was configured with role-based permissions, restricted access to identifiable information, and site-level visibility controls, enabling secure multi-site data entry while maintaining confidentiality and data integrity.

#### 2.1.2 Data instrument development and structure

**Figure 1:**
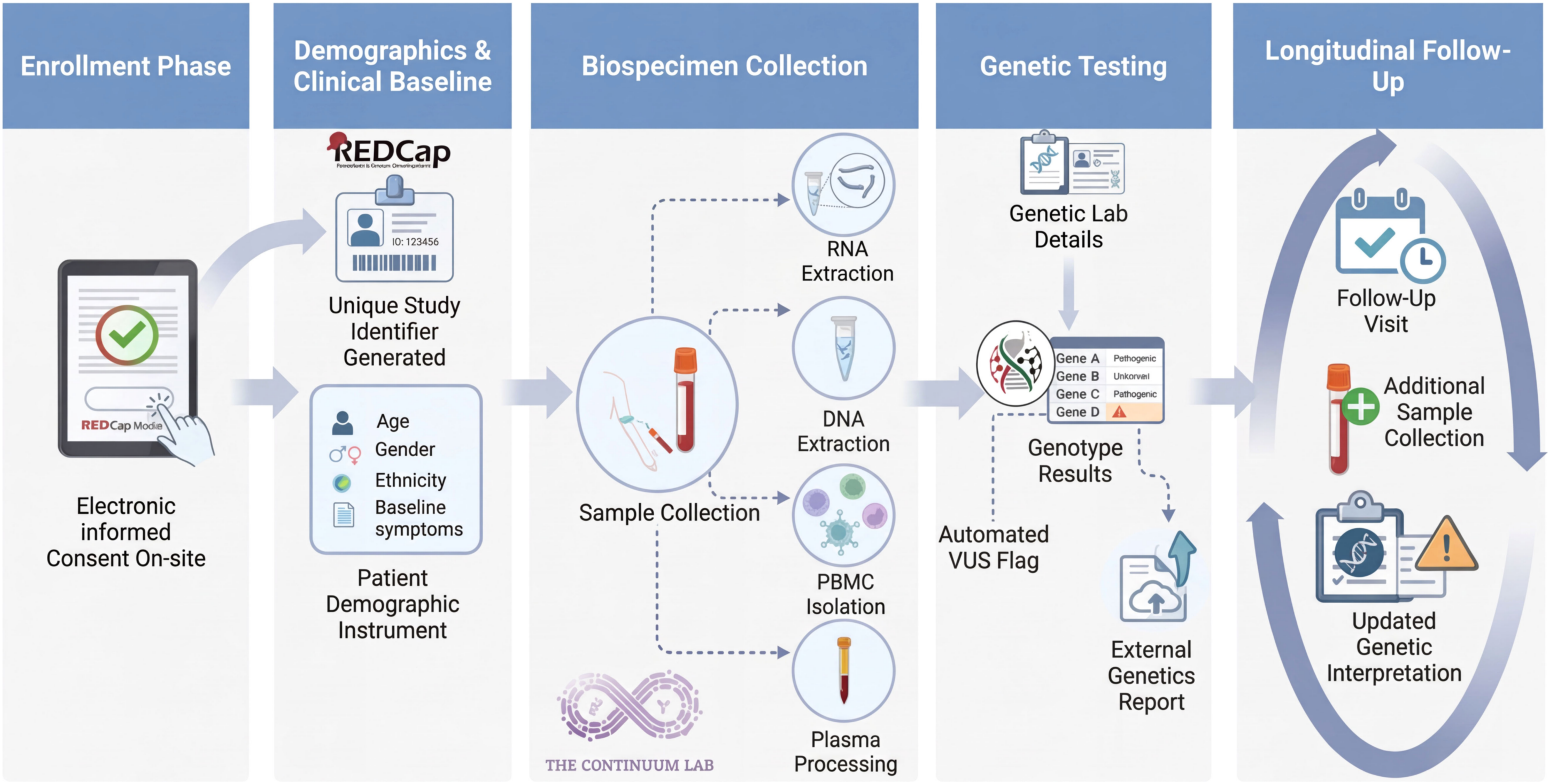
Stepwise REDCap longitudinal data collection workflow. The workflow includes (1) electronic informed consent and participant enrollment, (2) standardized demographic and clinical data capture with assignment of a unique study identifier, (3) peripheral blood collection and processing for DNA, RNA, PBMC, and plasma, (4) integration and interpretation of genetic test results with automated VUS flagging according to ACMG/AMP criteria, and (5) longitudinal follow-up with repeat sample collection and genetic reassessment of variants of uncertain significance (VUS).

The tool was organized into a modular instrument structure reflecting the clinical and laboratory workflow of patients with IEI. The Patient Demographics instrument recorded baseline demographic and clinical background information, including age, sex, nationality, presenting clinical manifestations, relevant immunological history, family history, consanguinity status, and pedigree information. The documented clinical features and relevant remarks are then mapped to standardized Human Phenotype Ontology (HPO) terms to facilitate variant analysis. The overall workflow and linkage structure of the REDCap IEI framework are illustrated in **Figure 1**. Peripheral blood is currently the only primary biospecimen collected and is processed for DNA, RNA, PBMC, and plasma preparation. Although other biospecimens, such as buccal swabs, saliva, bone marrow, and tissue specimens, are not currently included, the framework can be expanded to accommodate additional specimen types in future studies, subject to scientific requirements and ethical approvals. A summary of the instruments, representative variables, and embedded standardization features is provided in **Table 1**.

**Table 1:** Overview of REDCap instruments and standardized data capture features within the framework.

Instrument	Primary purpose	Representative variables	Data standardization features
Informed Consent	Record participant enrollment and consent	Participant ID, Consent date, Signed consent, Data sharing preference	Mandatory fields, e-Consent timestamp, Branching logic
Patient Demographics	Capture baseline participant information	DOB, Sex, Nationality, Phenotype, Age at diagnosis	Dropdown menus, Standardized terminology, Required fields
Sample Details	Track biospecimen collection and storage	Sample type, Collection date, Volume/count, Storage location, Sample ID	Standardized sample categories, Barcode linkage, Validation logic
DNA/RNA Extraction	Document nucleic acid extraction and QC	Extraction method, Concentration, Purity, Storage location	Standardized protocol selection, QC validation ranges
PBMC & Plasma Processing	Record immune cell and plasma processing	PBMC count, Viability %, Plasma volume, Freezing method	Validation logic, Protocol checkboxes, Controlled entries
Genetics Laboratory Details	Record sequencing workflow metadata	Platform, Assay type, Read length, Coverage metrics	Standardized assay/platform fields, Structured QC metrics
Genetic Results	Capture genomic variant findings	Gene, HGVS notation, Protein change, ACMG classification	HGVS validation, HUGO gene standardization, ACMG dropdowns
External Genetic Results	Integrate external genetic reports	External lab, Test type, Report date, Variant summary, VUS status	PDF upload fields, structured manual genomic harmonization, standardized test categories

**Figure 2:**
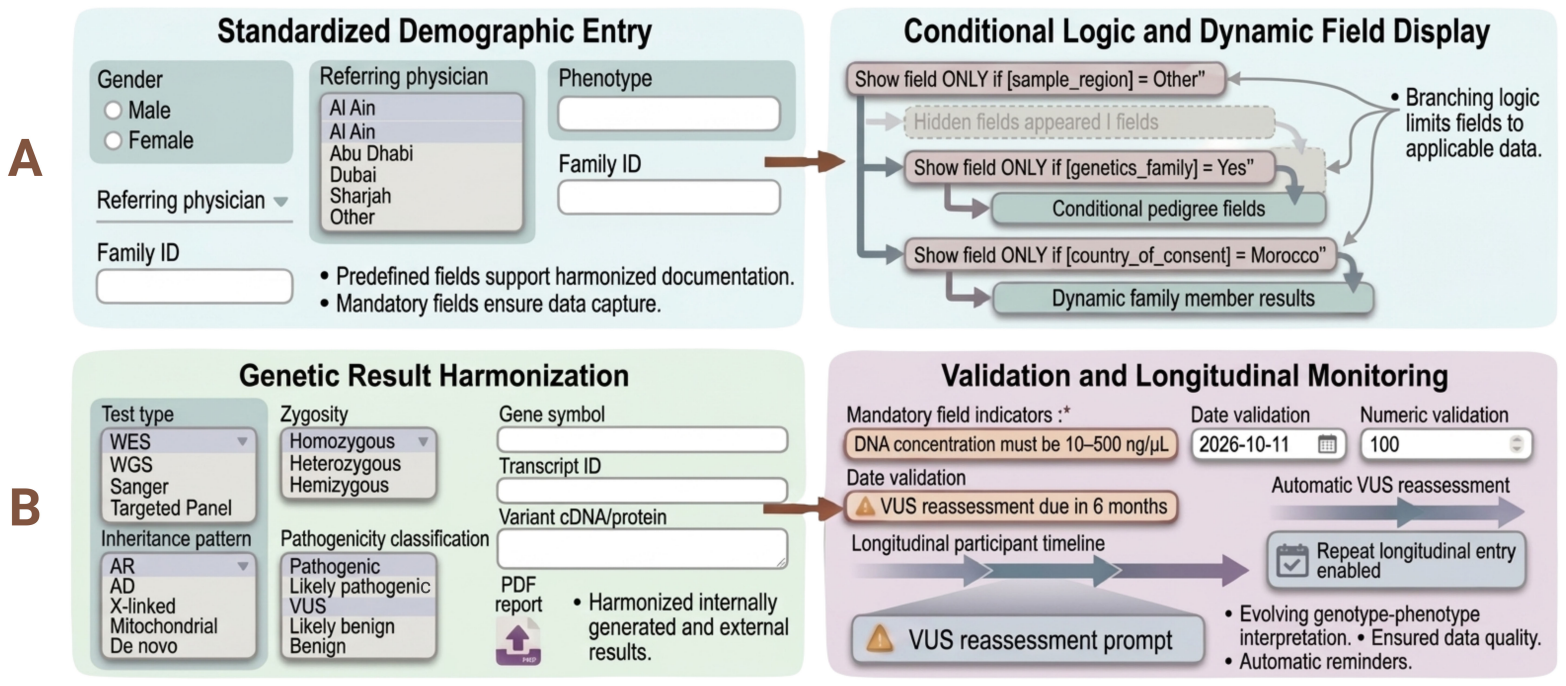
Embedded data standardization and validation features. **(A)** Standardized demographic data entry and conditional branching logic within REDCap to support harmonized and context-specific data capture. **(B)** Genetic result harmonization and longitudinal validation features, including standardized variant classification, automated checks, reminders, and reassessment tracking.

A standardized data dictionary was established prior to implementation, with variables structured using consistent naming conventions and predefined response options. Categorical fields were prioritized to support data harmonization. Required fields, range limits, and built-in validation rules were incorporated to reduce incomplete or inconsistent data entry. Conditional branching logic was implemented across instruments to display only contextually relevant fields. For example, laboratory extraction fields appeared only after documenting the corresponding sample type, and genetic interpretation fields were activated upon entry of sequencing results. This reduced unnecessary data entry, minimized user error, and streamlined workflows in both clinical and laboratory settings. The framework accommodates results generated through targeted gene panels, whole-exome sequencing, and whole-genome sequencing. Research-based sequencing is primarily performed in-house through the UAE University genomics facility, while external genetic investigations conducted as part of routine clinical care may also be incorporated. For in-house sequencing, VarSeq is used for variant annotation, filtering, and prioritization. Candidate variants are evaluated using OMIM, ClinVar, HGMD, population-frequency databases, in silico prediction tools, inheritance patterns, phenotype compatibility, and relevant literature. Variant classification is performed according to ACMG/AMP criteria and reviewed by a multidisciplinary team comprising genetics, immunology, genomics, and research personnel. For externally generated results, the original report is uploaded to REDCap, and key findings are entered into structured fields. Automated optical character recognition (OCR)-based extraction pipelines were not implemented but may represent a future development direction. Examples of embedded standardization, conditional logic, genomic harmonization, and longitudinal validation features implemented within the framework are shown in **Figure 2**.

#### 2.1.3 Unique identifier and record linkage

Each participant received a system-generated unique study identifier at enrollment within the informed consent instrument. Each participant identifier was assigned consecutively and configured to prevent duplication. For related participants, a unique family ID was generated within the laboratory and assigned to all recruited family members to enable linkage across records. Core demographic data were entered once and linked across all subsequent instruments, maintaining consistency while minimizing redundant entry and potential transcription errors. Specimen-level records were linked to the corresponding participant identifier, enabling integration of biospecimen processing data with associated clinical and genetic information.

### 2.2 Research and clinical application

#### 2.2.1 Longitudinal data capture

The framework was configured as a longitudinal project to allow repeated clinical and laboratory documentation over time. Repeated instruments were enabled for sample collection and genetic testing, allowing multiple entries per participant as new specimens were processed or genetic interpretations evolved. Within the Genetics Results instrument, structured fields were implemented to capture variant classification, including pathogenic, likely pathogenic, variant of uncertain significance (VUS), and benign categories. Records containing variants classified as VUS were automatically flagged using predefined logic, generating alerts for reassessment and enabling structured review workflows to support variant reclassification or updated clinical correlation.

In addition to longitudinal patient tracking, the platform supports both patient-level and cohort-level data review workflows. Individual participant records can be visualized through REDCap dashboards and event-based timelines, enabling longitudinal review of clinical, laboratory, biosepecimen, and genetic data across multiple study encounters. At the cohort level, REDCap filtering and querying tools enable aggregate review and the generation of structured summary datasets based on user-defined variables and study parameters. Exported datasets can subsequently be used for downstream analysis and visualization using external software platforms such as Microsoft Excel, R, and Graphpad Prism. Other longitudinal management modules are not currently operational and are proposed as part of a future Phase II expansion. Planned developments include therapeutic monitoring, treatment response assessment, adverse event tracking, HSCT/BMT follow-up, long-term clinical outcomes, and patient-reported quality-of-life measures. As the scope and sequence of Phase II will depend on project requirements, resource availability, ethical approvals, and participating-site readiness, a detailed implementation timeline has not yet been established, and development will proceed through a phased, milestone-based process.

#### 2.2.2 Clinical workflow integration and mobile consent

The data management tool was integrated into routine clinical and laboratory workflows. REDCap Mobile was configured to enable secure, tablet-based documentation of informed consent within clinic settings. Offline data capture functionality was enabled when required, with subsequent synchronization to the central database. Laboratory modules were structured to align with bench-side documentation practices, and conditional logic limited display of non-applicable processing fields.

#### 2.2.3 Ethics and data governance

The project operates under institutional ethical approval [Ref: DOH/ADHRTC/2025/1156]. Ethical safeguards were incorporated into the registry configuration, including restricted access to identifiable data, controlled export permissions, and site-level access segmentation. Identifiable information was limited to authorized clinical users, and analytical datasets were generated in de-identified format. Study identifiers were separated from clinical identifiers within exportable datasets to maintain confidentiality while preserving longitudinal linkage. Given the longitudinal nature of IEI and the potential for variant reinterpretation, structured informed consent addressed long-term data storage, possible reanalysis, and the return of clinically actionable findings. The tool operates within the legal framework of the United Arab Emirates, including Federal Law No. 4 of 2016, the Health Data Law of 2021, and the Federal Genomic Law of 2023, which regulate the protection, governance, and responsible use of human genomic data (15–17).

## 3 Results

### 3.1 Tool performance and system functionality

The integrated platform enabled coordinated documentation of enrollment, biospecimen processing, and genetic reporting within a unified longitudinal participant record. Participant-level records linked demographic information, laboratory procedures, and genetic findings, enabling traceability from specimen collection through molecular investigation. Genetic results were recorded within structured fields capturing gene-level findings, inheritance patterns, and variant classifications. The system also supported documentation of externally generated genetic results through secure upload of laboratory reports, allowing consolidation of all genetic findings within the participant record. In addition, the framework supports structured monitoring of genetic testing workflows. Once a specimen is dispatched for sequencing, testing status is documented and tracked until results are recorded in the participant’s longitudinal record. Variants classified as variants of uncertain significance are automatically flagged within the system, triggering a structured reassessment workflow six months after initial documentation. This monitoring mechanism enables systematic review and documentation of variant reclassification as new evidence becomes available, which is particularly relevant in IEI, where genotype–phenotype relationships frequently evolve.

### 3.2 Field research implementation

#### 3.2.1 Participant enrollment and biospecimen documentation

During the current implementation period, 21 participants were enrolled, with the first recruitment recorded on 25 September 2025. The enrolled participants represented a broad spectrum of suspected or confirmed IEI presentations, including combined immunodeficiencies, predominantly antibody deficiencies, immune dysregulation disorders, autoinflammatory conditions, and defects of phagocyte function. These examples are provided to illustrate the range of clinical presentations accommodated by the framework rather than to present a formal analysis of the enrolled cohort.

Enrollment generated a unique study identifier linking all clinical, laboratory, and genetic data. Participant information was maintained within longitudinal records to enable ongoing documentation of clinical reassessments and biospecimen collections. A total of 21 biospecimen records were documented during this period. Blood samples underwent standardized laboratory processing, including RNA extraction, DNA extraction, PBMC isolation, and plasma preparation. DNA samples were stored at −80 °C for confirmatory sequencing and potential reanalysis, while RNA, PBMC, and plasma specimens were preserved for downstream transcriptomic, cellular, and biomarker analyses.

#### 3.2.2 Multi-site implementation and operational performance

The framework was implemented as a centralized expandable multi-site platform and is currently used by two participating centers. Clinical, laboratory, and genetic documentation are captured within a shared system instance using identical instrument structures across sites, ensuring standardized data collection. Site-level access controls maintain appropriate record visibility while preserving a unified data architecture. Consistency across participating sites was supported through the use of a centralized REDCap instance with harmonized instrument configuration, standardized data dictionaries, predefined categorical fields, mandatory variables, and embedded validation rules designed to minimize structural discrepancies and inconsistent data entry. Additional quality-control measures included role-based user permissions, restricted modification privileges, regular data review procedures, and structured training sessions for clinical and research personnel involved in data collection and management. No structural discrepancies requiring reconciliation between participating sites have been identified since deployment. Embedded validation rules promote data completeness at the point of entry, while integration of laboratory and genetic modules strengthens traceability from specimen acquisition to molecular results.

## 4 Discussion

Inborn errors of immunity (IEI) present distinctive challenges for clinical documentation because diagnosis often evolves as phenotypes change, laboratory investigations are repeated, and genetic findings are reinterpreted. In routine clinical practice, however, relevant data are frequently dispersed across clinic notes, laboratory records, and external genetic reports, making longitudinal interpretation difficult. The framework described here was designed to address this challenge by integrating clinical phenotyping, biospecimen documentation, and genetic interpretation within a single evolving record that reflects the diagnostic trajectory of IEI.

Several electronic data capture platforms are used in clinical research, including REDCap, OpenClinica, Castor EDC, and ClinCapture. These platforms differ in cost models, flexibility of form design, longitudinal data structures, and capacity to integrate diverse clinical and laboratory datasets, as summarized in **Table 2** (3–6, 18). Among these systems, electronic data capture platforms such as REDCap are widely used in clinical research because of their flexible instrument design, secure institutional hosting, and support for distributed multi-site data entry (3, 4). In the present framework, REDCap was configured to support an integrated clinical–laboratory–genetic workflow, enabling structured documentation of enrollment, biospecimen processing, and genetic testing within a longitudinal participant record. This structure enables traceability from specimen collection to molecular results and allows iterative updates as new data emerge.

**Table 2:** Key characteristics of commonly used electronic data capture platforms.

Feature	REDCap	OpenClinica	Castor EDC	ClinCapture
Primary use	Academic research, cohort studies	Clinical trials and research studies	Clinical trials and observational studies	Clinical trials
Cost model	Free for consortium institutions	Open-source core; enterprise licensing	Commercial subscription	Commercial subscription
Hosting	Institution-hosted	Cloud or on premises	Vendor-hosted cloud	Vendor-hosted cloud
Form design	Highly flexible investigator-defined instruments	Protocol-driven forms	Template-based forms	Structured form builder
Longitudinal data capture	Native events and repeating instruments	Visit-based trial structure	Visit-based study design	Scheduled visits
Multisite collaboration	Role-based permissions, Data Access Groups	Site-level permissions	Centralized study management	Multicenter trial configuration
Ability to combine clinical, laboratory, and genetic data	Flexible integration within a single participant record	Primarily structured for trial datasets	External integrations supported	Structured clinical datasets

Rare disease initiatives such as RareLink and GenIA have further demonstrated the growing importance of integrated platforms capable of harmonizing phenotypic and genomic data in rare immune disorders for linking clinical phenotype and genomic data across distributed datasets (10, 19). Building on this concept, the approach described here incorporates structured documentation of laboratory workflows and genetic testing, including mechanisms to monitor reassessment of VUS, which is particularly relevant in IEI, where genotype–phenotype relationships continue to evolve. Although primarily designed to support clinical and laboratory workflows, the modular architecture could facilitate future cross-registry data exchange through the adoption of standardized phenotype ontologies and interoperability frameworks similar to those implemented in RareLink (19). At present, the platform functions mainly as a structured data capture system and does not include automated genomic interpretation capabilities such as variant annotation or direct integration with external genomic databases. Previous REDCap-based multi-site infrastructures, such as the mesothelioma platform described by Rashid et al., demonstrated the utility of REDCap for federated biobanking and cohort discovery in rare disease research. In contrast, the present framework was designed for longitudinal IEI-focused clinical and genomic data integration, including structured phenotype capture and variant reassessment workflows across participating centers. Future expansion of our framework may include broader federated collaboration, integrated biobanking capabilities, and cohort-level research infrastructure.

To our knowledge, detailed descriptions of REDCap-based systems integrating clinical documentation, biospecimen processing, and genetic data within a longitudinal framework for inborn errors of immunity remain limited. The approach presented here supports traceability across the diagnostic workflow while enabling standardized data capture across participating sites. The framework has been implemented across two centers, with ongoing enrollment. Current implementation is supported through a distributed operational model involving trained research personnel and designated clinical residents working alongside supervising clinical immunology investigators at participating sites. Patients meeting the inclusion criteria are identified during routine clinical care, while research assistants support consent coordination, structured REDCap data entry, biospecimen documentation, and longitudinal follow-up. This model was designed to minimize additional documentation burden on treating clinicians while maintaining standardized prospective data collection across sites. Institutional research infrastructure and participating site personnel currently support implementation. At present, the framework primarily functions as a longitudinal IEI research and data management system rather than a routine clinical documentation platform. There are currently limited implemented national or regional IEI-focused registries or standardized longitudinal data infrastructures within the UAE/MENA region. While collaborative initiatives such as the MENAT-IEI Research Consortium have recently been proposed, large-scale integrated registry implementation and data harmonization platforms remain limited (11). The present framework was therefore developed to support future multi-center clinical, laboratory, and genomic data integration within the UAE/MENA region.

REDCap was selected in part because of its scalability and ability to support implementation across institutions with varying technical infrastructure and financial resources. Following initial implementation, broader dissemination will be pursued through regional and national collaborations using standardized deployment protocols aligned with institutional governance requirements and UAE data protection regulations (15–17). Future expansion will be supported through structured user training initiatives, harmonized instrument configuration, and role-based institutional access to facilitate consistent multi-site adoption.

Currently, enrollment primarily occurs following clinical suspicion or identification of IEI during routine clinical evaluation. Future development may include interoperability with regional electronic health record (EHR) systems to support earlier identification of patients with suspected IEI and facilitate prospective enrollment workflows. Integration of computational screening approaches, including machine learning-assisted patient stratification models, may further support early detection of potentially undiagnosed cases. However, the platform is intended to function alongside existing clinical information systems as a complementary longitudinal research and data management tool rather than a replacement for institutional EHRs. While EHR systems primarily support routine clinical documentation and often vary substantially across institutions, the present framework enables harmonized longitudinal integration of clinical, laboratory, and genomic data within a structured research-oriented environment. The platform supports standardized exportable datasets, centralized genomic tracking, longitudinal variant of uncertain significance (VUS) reassessment workflows, and cross-site research harmonization, which are not routinely optimized within fragmented EHR-based documentation systems. In addition, the framework facilitates downstream statistical analyses, genotype–phenotype correlation studies, biospecimen traceability, and future collaborative rare disease research initiatives through the generation of research-ready datasets suitable for downstream analysis and visualization.

The present study should therefore be interpreted as an early implementation report intended to demonstrate the operational capacity and functionality of the framework. The enrollment of 21 participants and documentation of their associated biospecimens confirm that the platform is active and capable of prospectively linking clinical, laboratory, biospecimen, and genetic information within longitudinal records. However, the present report was not designed to characterize the enrolled cohort or evaluate diagnostic yield, genotype–phenotype associations, variant resolution, treatment outcomes, or long-term clinical impact. These aspects will require a larger cohort, continued enrollment, and longitudinal follow-up and will be addressed through subsequent dedicated analyses.

As the framework evolves and the network expands, the modular architecture of the platform allows incorporation of newly identified genes, updated diagnostic classifications, and emerging clinical insights without substantial modification of the underlying data structure. Alignment with classifications from the International Union of Immunological Societies (IUIS) could further support standardized categorization of IEI diagnoses, while future development will incorporate longitudinal therapeutic monitoring modules, including treatment response assessment, adverse event tracking, hematopoietic stem cell/bone marrow transplantation follow-up, immune reconstitution monitoring, long-term clinical outcome evaluation, and patient-reported quality-of-life measures. Overall, this REDCap-based data model provides a practical framework for prospective genotype–phenotype data collection in IEI research and may support future collaborative data sharing.

## Data Availability

The original contributions presented in the study are included in the article/supplementary material. Further inquiries can be directed to the corresponding author.
